# Spreading depression as a preclinical model of migraine

**DOI:** 10.1186/s10194-019-1001-4

**Published:** 2019-05-02

**Authors:** Andrea M. Harriott, Tsubasa Takizawa, David Y. Chung, Shih-Pin Chen

**Affiliations:** 1 Neurovascular Research Lab, Department of Radiology, Massachusetts General Hospital, Charlestown, MA USA; 2 Department of Neurology, Massachusetts General Hospital, Boston, MA USA; 3 Department of Neurology, Keio University School of Medicine, Tokyo, Japan; 4 Institute of Clinical Medicine, National Yang-Ming University, Taipei, Taiwan; 5 Brain Research Center, National Yang-Ming University, Taipei, Taiwan; 6 Division of Translational Research, Department of Medical Research, Taipei Veterans General Hospital, Taipei, Taiwan; 7 Department of Neurology, Neurological Institute, Taipei Veterans General Hospital, Taipei, Taiwan

**Keywords:** Migraine, Aura, Spreading depression, Optogenetics

## Abstract

Spreading depression (SD) is a slowly propagating wave of near-complete depolarization of neurons and glial cells across the cortex. SD is thought to contribute to the underlying pathophysiology of migraine aura, and possibly also an intrinsic brain activity causing migraine headache. Experimental models of SD have recapitulated multiple migraine-related phenomena and are considered highly translational. In this review, we summarize conventional and novel methods to trigger SD, with specific focus on optogenetic methods. We outline physiological triggers that might affect SD susceptibility, review a multitude of physiological, biochemical, and behavioral consequences of SD, and elaborate their relevance to migraine pathophysiology. The possibility of constructing a recurrent episodic or chronic migraine model using SD is also discussed.

## Background

Spreading depression (SD) or, more appropriately, spreading depolarization, is a slowly propagating wave of near-complete depolarization of neurons and glial cells spreading over the cortex at a speed of ~ 3–5 mm/min [1, 2]. SD is characterized by a profound change in transmembrane ion gradients and loss of all spontaneous or evoked synaptic activity and action potentials, resulting in depression of electrocortical signals [2]. The exchange of intracellular and extracellular components during SD is composed principally of a large influx of Na^+^, Ca^2+^, and water, and efflux of K^+^, H^+^, glutamate, and adenosine triphosphate (ATP) [2–6]. The rise of extracellular K^+^, rather than glutamate diffusion, may be the leading event that diffuses to and depolarizes adjacent cells [6]. Since the original publications of Leao [1, 7], experimental SD has been recorded in the cortices of both lissencephalic (e.g. rodents or rabbits) [8] and gyrencephalic (e.g. feline or swine) cortex [9–11]. A link between SD and migraine pathogenesis has been hypothesized for decades [12], in particular the visual aura [13, 14] and more recently the migraine headache.

### Aura phenomenology and SD

Migraine with aura occurs in 30–40% of patients diagnosed with migraine and is most commonly a visual disturbance. The visual disturbance can be variable and include fortification spectra, sparkling or shimmering colored dots and blobs, and scotoma [15–17]. While visual symptoms are the most commonly described aura event of migraine, other auras including sensory and speech disturbance have been described. In one study visual aura occurred in 98% of those with migraine with aura, while sensory symptoms including paresthesias and hypoesthesia occurred in 36% and dysphagic symptoms in 10% [18]. In those with more than one aura symptom, the onset of the second or third aura symptom seem to follow the first or second aura symptom in succession, i.e. the additional aura symptom starts subsequent to the start of the preceding aura symptom. In those with two aura symptoms, the second symptom started after the onset of the first 66% of the time. In those with three aura symptoms, the third symptom started after the onset of the second 82% of the time [19].

There are several clinical studies supporting SD as the likely mechanism involved in the aura event of migraine which has been the topic of multiple well written review articles. In early depictions of migraine aura, Lashley postulated that the positive symptom resulted from a region of cortical hyperexcitability while the scotoma likely related to an area of diminished cortical activity spreading across visual cortex. It was further surmised based on the rate of spread that the velocity of this electrical event was about 3 mm/minute. The event of cortical SD (CSD) recorded by Leao, having congruent temporal pattern and spread, raised the possibility that SD was the underlying electrophysiological event of migraine aura [17, 20]. Several clinical studies have since supported this relationship between migraine aura and SD. Both SD and the migraine aura phase are associated with pronounced oligemia as noted in multiple Xenon based and single photon emission computed tomography imaging studies [17, 21]. In one study examining functional magnetic resonance blood oxygen level dependent (BOLD) signaling during migraine aura, increased BOLD signal propagated across visual cortex retinotopically coincident with movement of the aura a rate resembling SD. This further buttressed the causal relationship of Leao’s SD to migraine aura [13, 22]. Symptoms other than the visual disturbance suggest that brain regions outside of striate cortex may be involved in migraine with aura and possibly affected by the spread of CSD [23] though this has not been confirmed in human studies. While direct clinical evidence that SDs are causally associated with sensory and other non-visual aura symptoms is limited, experimental SDs can be generated from various anterior and posterior cortical brain regions highly suggestive of SD as a neurobiological phenomenon responsible for these aura symptoms. Some migraine with aura sufferers experience sensory and visual symptoms simultaneously (i.e. without succession) raising the possibility that in addition to spread, SD could be generated in multifocal regions simultaneously [18, 19].

### SD in relation to migraine headache

There are arguments both for and against a temporal relationship between the migraine aura and headache. While some will experience aura without headache, most migraine attacks with aura are accompanied by headache (91%) [18]. While the headache can occur before or simultaneous with the aura event, the headache in most cases (78%) occurred after the onset of aura either during the aura phase (28.7%), at cessation of the aura (12.1%) or a period after aura cessation (37.6%) [18].

Activation of the trigeminovascular system (TVS) is critical to migraine pathogenesis [24–30]. CSD may be a key CNS trigger for TVS activation [31]. CSD can activate perivascular trigeminal afferents and evoke a series of cortico-meningeal and brainstem events consistent with the development of headaches [32–36]. CSD leads to increased expression of the immediate early gene product c-FOS in the trigeminal nucleus caudalis (TNC), sterile neurogenic meningeal inflammation mediated by trigeminal collateral axons, and dilatation of the middle meningeal artery via the trigemino-parasympathetic reflex [32]. Single unit recording studies showed that CSD can lead to delayed and long-lasting activation of meningeal nociceptors in the trigeminal ganglion [33] and central TVS neurons in the TNC [34]. The precise mechanism that triggers TVS activation has yet to be elucidated, but SD can cause the release of inflammatory and diffusible substances in the cortex including prostanoids, nitric oxide, ATP, and K^+^ [37]. In addition to glutamate release and collapse of ionic gradients; SD can activate purinergic receptors and pannexins, large pore channels whose stimulation can produce brain inflammation [36, 38]. In fact, SD can increase brain cytokine release and astroglial activation. In addition to local cortical responses to SD, diffusible substances may reach the overlying meningeal surface and potentially activate trigeminal neuropeptide containing axons leading to peripheral and central release of calcitonin gene related peptide (CGRP) [32, 39, 40]. This and other mechanisms may be involved in meningeal inflammation and peripherally and sensitization of TNC neurons centrally. The stimulation of these nociceptive pathways may be involved in the pain of migraine. That SD can trigger a series of events likely involved in the headache phase of migraine provides a plausible biological link between SD generation and migraine pain that may not be necessary for the generation of migraine pain but in some cases, it may be sufficient. Hence, SD may be not only the physiological substrate of migraine aura, but also a potential cause of headache. Although it is a matter of debate whether migraine patients without aura have asymptomatic SD, a recent study suggests that the visual percept of aura can be clinically silent [41]. While speculative, it is possible that some migraine patients without a perceived aura might have SD-like activities propagating through ineloquent cortex.

### Why consider SD model for the study of migraine

There are limitations to the approach of using SD as a model to study mechanisms that may be associated with migraine. Much like other models, it is one component part of a complex heterogenous disease process involving genetic, sex dependent, hormonal and environmental factors. Therefore, like other models including meningeal application of exogenous inflammatory substances, SD does not encompass all the disease complexity of migraine. However, it does allow for the examination of alterations in cortical and subcortical brain excitability and nociceptor activation. There are several shortcomings of the SD model that are detailed below including the invasive conventional methods previously employed which may resemble a model of injury as opposed to migraine. While there are concepts that challenge the link between SD and the headache of migraine [42], including the variable onset of headache following aura symptoms, aura without headache and several incongruent preclinical observations; the evidence for a plausible causal relationship of SD to trigeminal nociceptor activation and therefore likely pain remain convincing. To the extent that SD can activate dural afferents and second order trigeminovascular neurons [40, 43], increase neuropeptide release and alter pain behavior [44]; it is a reasonable experimental model to investigate SD mechanisms involved in migraine with aura. Moreover, since trigeminal activation is a critical component of migraine pain, SD mediated activation of trigeminal neurons and peripheral release of neuropeptides may link the aura of migraine with the pain experienced during an attack.

**Fig. 1:**
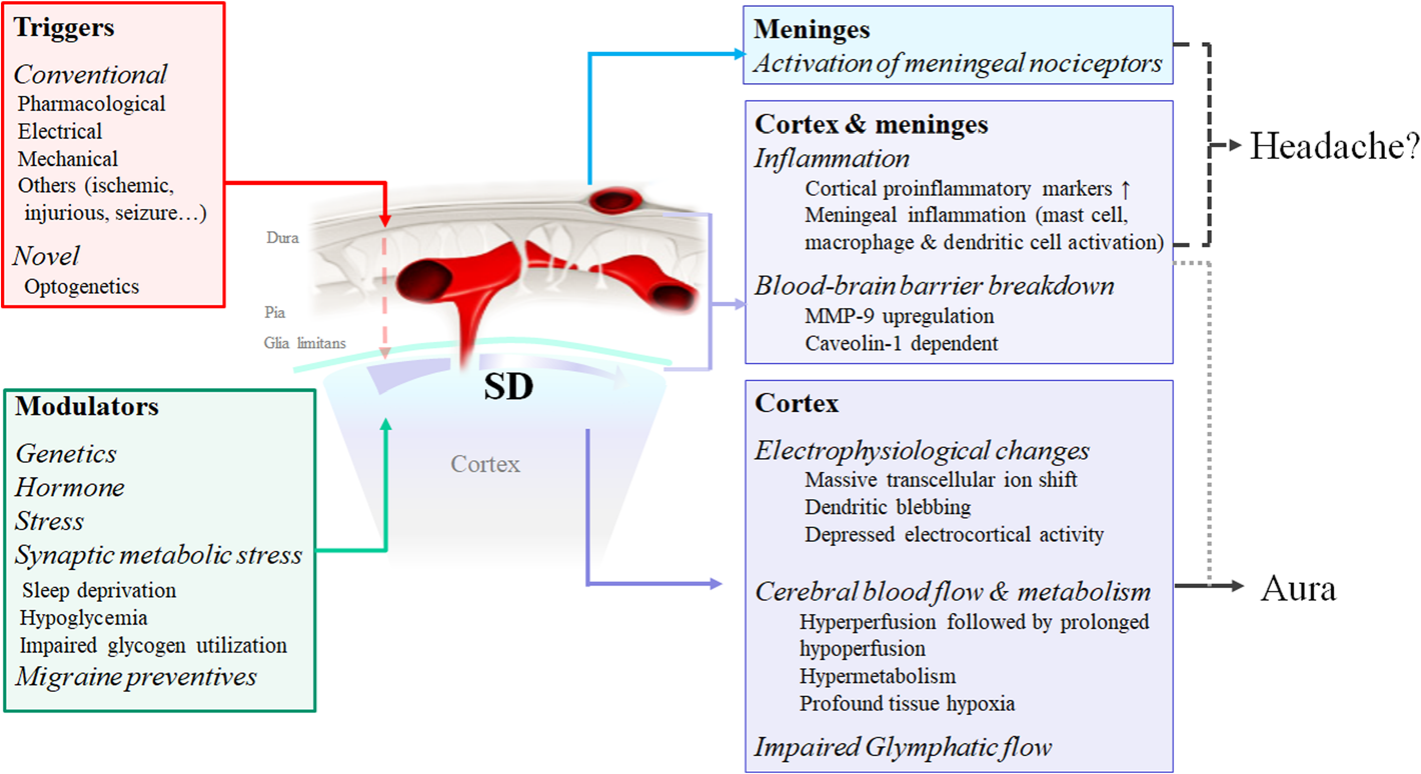
Triggers, modulators and consequences of spreading depression in experimental animal models

In this review, we summarized the currently known experimental models of SD, reviewed the triggers, modulators and consequences of SD, and elaborate their relevance to migraine (Fig. 1).

## SD susceptibility and its clinical translatability

Although direct electrophysiological evidence of SD in patients with migraine is still lacking, SD has attracted considerable attention for its translational relevance [45]. Experimental models of SD have recapitulated multiple clinical characteristics of migraine in human subjects and have been used to explore the basic mechanisms, genetic and hormonal modulators, and potential physiological or pharmacological inhibitors of migraine [45, 46]. SD susceptibility is one measure used to examine such relationships. The study of SD susceptibility involves the exploration of the vulnerability of brain tissues to occurrence, propagation and recurrence. Depending on the detection method, various SD susceptibility measurements have been used to study the physical and biochemical attributes of SD [45, 47]. The stimulus intensity used to evoke SD is one of the most relevant attributes of SD susceptibility. Depending on the modality, stimulus intensity threshold can be measured in electrical charge intensity, volume or concentration of a depolarizing agent, or mechanical pressure. Another commonly measured SD susceptibility attribute is the frequency of SDs triggered during continuous topical application of suprathreshold concentrations of depolarizing agents. Propagation speed is also a reliable measure of SD susceptibility, and has good correlation with the threshold and frequency of SD [47]. Other SD attributes such as amplitude and duration do not correlate well with susceptibility [47]. Observations of SD susceptibility suggest sex and genetic variables associated with migraine alter the brains vulnerability to SD generation. For instance, there seems to be a reduced threshold for SD in female mice [48], which fits with the observation that migraineurs are predominantly female. *Cacna1a*^R192Q^ knock-in (KI) mice that carry the human pathogenic familial hemiplegic migraine 1 (FHM1) R192Q mutation express an abnormally low SD threshold [49], consistent with the notion that the brains of migraineurs are hyperexcitable [50, 51]. In addition, clinically efficacious migraine prophylactic drugs, despite their different mechanisms, all inhibit SD susceptibility in vivo [47, 52, 53]. Aging is also known to modulate SD susceptibility, which declines with senescence. However, despite reduced SD susceptibility, the consequences of SD recurrence in the older brains are more detrimental than that in younger brains [54].

## Experimental methods of SD induction

### Conventional methods

Experimentally-evoked SD in normally metabolizing brain tissue requires intense depolarizing stimuli. An increase of extracellular K^+^ above a critical threshold concentration (12 mM) in a minimum volume of brain tissue (1 mm^3^) is estimated to be the minimal requirement to provoke SD in rodents [55, 56]. A variety of stimuli, spanning pharmacological, electrical, and mechanical modalities, have been used to induce SD [2, 57, 58]. Each has its own caveats and possibly differential mechanisms [47, 59].

#### Pharmacological induction

The most commonly used agent to evaluate susceptibility to SD, KCl, may be administered as a suprathreshold concentrated solution, escalating concentration or volume of brief pulse KCl solution, or KCl crystals. Glutamate [60] or N-methyl-D-aspartate (NMDA) receptor agonists [61], Na^+^/K^+^–ATPase inhibitors (ouabain) [62, 63], and endothelin-1 [58, 64] have also been used to evoke SD. The administration route of these depolarizing agents may include topical application or intraparenchymal injection. These agents are typically applied through burrhole craniotomy on the meningeal surface or the surface of the exposed cortex or on thinned skull allowing them to diffuse to the meningeal and cortical surface below. Like KCl, steps of escalating concentrations or continuous suprathreshold concentration of these depolarizing agents can be used to evaluate the threshold to evoke SD or the frequency of SD.

#### Electrical induction

Electrical stimulation is another reliable method used to assess SD susceptibility [47, 52, 65, 66]. Usually, it is delivered as escalating steps of single square wave pulses or as high-frequency train stimulation with escalating intensity and/or duration. The minimal electrical volume required to trigger SD is defined as the electrical threshold. The condition of the cortex, the age of the animals, electrode properties, and the contact between electrodes and tissues are critical components determining the final threshold [47, 54, 67].

#### Mechanical induction

Mechanical stimuli, especially needle prick, provide an intense depolarization sufficient to evoke SD. However, it is difficult to determine a threshold of mechanically-induced SD, and the reproducibility varies between operators and settings. Direct traumatic injury to the cortex and related bleeding are also important concerns. The mechanism of mechanical stimuli-evoked SD might be related to Na^+^ channels [68], and the related cerebral blood flow changes might be associated with AMPA and GABA receptors [69].

#### Other conventional methods

Some supra-physiological or pathological triggers of SD have been observed in vivo. Experimental ischemia, hypoxia, and microemboli have been reported to trigger SD [70–76], and may be helpful for understanding the pathogenesis of migraine-stroke comorbidity. These ischemia-evoked SDs may be triggered by supply-demand mismatch transients [77], circle around and enlarge the ischemic lesions [77, 78], and be suppressed by migraine preventive drugs [70]. SDs in these pathological conditions might be a cause of headaches associated with cerebral ischemia [46]; however, these headaches, by definition, should not be classified as migraine.

### Novel optogenetic methods to trigger SD

The conventional methods to induce SDs discussed above have been critical in advancing our current understanding of the role of the phenomenon in human disease. However, inferences about the role of SD in inflammation, for example, could be confounded by the invasive nature of conventional SD induction methods. Therefore, a non-invasive approach for the induction of SDs could be a useful complement to conventional methods.

Optogenetics technology enables non-invasive, real-time stimulation of targeted brain cells, and provides the potential for detailed, precise insight into disease mechanisms in awake animals [79, 80]. Investigators have recently developed such a non-invasive approach by utilizing transgenic optogenetic mouse lines where a light-responsive ion channel called channelrhodopsin-2 (ChR2) is expressed in excitatory cortical neurons under the Thy1 promoter [81–84]. This new optogenetic approach allows for the controlled induction of SDs through intact skull using 470 nM blue wavelength light stimulation. Optogenetic SDs can be induced as single events, repeated to determine the impact of recurrence and produced in both anesthetized or awake and behaving animals. Importantly, the technique enables longitudinal study of SDs over the course of weeks without brain injury confounders caused by invasive SD induction and detection methods. Optogenetic SD induction can be detected with multiple methods including optical intrinsic signal (OIS) imaging. However, when simultaneously examining SD detection using this method in combination with other techniques (electrode recording, laser speckle imaging, and laser doppler flowmetry); the fidelity and reproducibility of the response detected with OIS was indeed comparable to more invasive methods like electrode recording [82].

### Spontaneous SD and physiological triggers

#### Spontaneous SD in wild-type and genetically-modified animals

Spontaneous SD is defined as SDs detected without direct provocation in the absence of acutely applied methods of induction (i.e. pinprick trauma, KCl application, or electrical stimulation). Perhaps limited by the techniques of in vivo recording in awake animals and the high threshold of SD in unprovoked healthy cortical tissue, there is little evidence of spontaneous SD in wild-type animals. Most of the literature reporting so-called spontaneous SD involve SDs in or around unhealthy cortical tissue. Genetically modified animals, including transgenic mice expressing familial hemiplegic migraine 1 (FHM1, *CACNA1A*) [49, 85–87], FMH2 (*ATP1A2*) [88], cerebral autosomal dominant arteriopathy with subcortical infarcts and leukoencephalopathy (CADASIL, *NOTCH3*) [89], and familial advanced sleep phase syndrome (FASPS, *CSNK1D*) [90] mutations, have higher susceptibility to SD. However, there is also scarce evidence suggesting that SD can occur spontaneously in the hyperexcitable brains of these transgenic mice.

#### Physiological SD triggers

##### Stress, sleep deprivation and hypoglycemia

Stress and its let-down have been shown to be important triggers of migraine [91, 92]. Stress may increase cortical excitability by increasing extracellular glutamate or corticotropin-releasing hormone. In a study using Swiss albino mice, SD threshold was reduced by acute and chronic stress as well as by central noradrenergic denervation [93]. However, in another study in which 14-day social defeat stress and 40-day chronic variable stress were introduced to male C57Bl/6 mice, no difference in SD frequency or velocity was seen [94]. In another study, acute stress (20-min and 3-h restraint stress) did not influence SD susceptibility in FHM1 transgenic mice or wild-type mice [95]. In contrast, exogenous administration of corticosterone increased SD frequency exclusively in mutant mice [95]. Further studies designed to dissect the complex biological stress responses are needed to resolve the inconsistency across studies. Although stress or its related hormones might affect SD susceptibility, it is not yet known whether spontaneous SD occurs upon acute or chronic stress or its let-down.

Sleep deprivation, hypoglycemia, and impaired glycogen utilization are important metabolic stresses to synapses. A common trigger of migraine, sleep deprivation, is known to limit the capacity of neurons to maintain low concentrations of extracellular glutamate and K^+^ during sustained excitatory transmission, which may be mediated by impaired glycogen utilization [96]. A recent study showed that sleep deprivation and impaired glycogen breakdown led to synaptic metabolic stress and lower SD threshold, which could be reversed by supplying a glycogen-derived energy substrate (i.e. glucose or lactate) [96]. Consistent with these findings, systemic hyperglycemia was found to elevate the electrical SD threshold and reduce the frequency of KCl-induced SDs [97], that is, to make the brain more resistant to SD. It would be interesting to see if combining these synaptic metabolic stressors could evoke spontaneous SD in either wild-type or genetically modified animals.

### Peripheral and central consequences of SD

#### Peripheral consequences of SD involving the meninges and trigeminal nociceptors

##### Meningeal inflammation

Meningeal vasodilation, plasma protein extravasation, and immune cell activation have all been observed in in vivo rodent SD models [32, 36, 98]. A study of middle meningeal arterial blood-flow monitoring after SD, using laser speckle-contrast imaging, demonstrated blood-flow increase from 5 min to 45 min after SD, accompanied by vasodilation. Plasma protein extravasation in dura was observed after CSD, using whole-mount preparation of dura matter after intravenous injection of horseradish peroxidase. Significant perivascular leakage was detected, and this leakage could be suppressed with application of a substance P (a.k.a. neurokinin-1) receptor antagonist [32]. In contrast, another study showed that SD does not alter dural plasma extravasation as measured by bovine serum albumin-coupled fluorescein [99].

With respect to meningeal immune cells, characteristics of dural mast cells after SD have been assessed by methylene blue staining. The percentage of degranulated dural mast cells increased significantly 30 min after SD [36]. There is additional evidence using two-photon microscopy that SD produces pial and later dural macrophage activation and increased pial dendritic cell mobility. The timing of activation of these peripheral immune cells is speculated to relate to the differing temporal relationship of the headache to the aura [98].

##### BBB breakdown

SD has been found to alter the permeability of BBB by activating brain matrix metalloproteases [100]. Levels of metalloprotease-9 increased in cortex beginning 3–6 h after SD, reaching a maximum at 24 h and persisting for at least 48 h [100]. .Interestingly, MMP-9 level was also found to be higher in patients with migraine than controls [101]. Recently, SD-induced BBB permeability to water and large molecules was found to be mediated by increased endothelial transcytosis, which starts between 3 h and 6 h and lasts for 24 h after SD [84]. This SD-induced BBB disruption and endothelial transcytosis is dependent on caveolin-1 and rho-kinase 2. Endothelial tight junctions, pericytes, and basement membrane, in contrast, remain preserved after SDs. A recent study in awake rats also found that cortical BBB leakage begins 0.5 h after induction of SD and resolves within 6 h, without altering expression of the tight junction proteins occludin or claudin-5 [102]. While SD mediated meningeal inflammation and BBB breakdown have been demonstrated in rodents, the role of these mechanisms in migraine pathogenesis remain unclear. It is unknown if BBB breakdown is only a consequence of inflammation or if it may play a contributory role in trigeminal pain or other associated features of migraine. On the other hand, BBB breakdown may have direct implications for the access of drugs to centrally located targets during the migraine attack. If these changes are transient following SD, it may provide a time window during aura in which may enhance CNS penetration of migraine specific medications like triptans or CGRP antagonists.

##### Activation of meningeal nociceptors

Single unit recordings have shown that cortical SD can lead to delayed and long-lasting activation of meningeal nociceptors in the trigeminal ganglion [33] and central trigeminovascular neurons in the TNC [34]. Although multiple SDs are generally induced during recordings in animal studies, a single cortical SD is sufficient to elicit persistent activation of meningeal nociceptors [35]. Two patterns of prolonged nociceptor activation—biphasic activation (brief activation around SD induction followed by delayed, persistent activation, primarily in the Aδ population) and persistent activation with delayed onset (in C unit population)—were observed following SD [35]. SD-evoked prolonged activation of meningeal nociceptors might be related to ongoing basal activity or the number of receptive fields, rather than to the inflammatory and ATP chemosensitivity of the neurons; SD-evoked activation and mechanical sensitization of meningeal afferent responses was dissociated from SD-evoked metabolic perturbations [103]. SD was also found to evoke a delayed meningeal afferent mechanosensitization, which might explain nociceptive processes that underlie the worsening of migraine headache in conditions associated with transiently increased intracranial pressure [104]. Recently, a humanized monoclonal anti-CGRP antibody Fremanezumab was found to inhibit SD-evoked activation of high-threshold neurons but not wide-dynamic range trigeminovascular neurons in the TNC [40]. This effect was mediated predominantly via thinly myelinated Aδ fibers rather than unmyelinated C meningeal nociceptors [105]. However, a CGRP receptor antagonist BIBN4096 inhibited prolonged meningeal afferent activation evoked by brief local K^+^ stimulation but not SD-induced afferent sensitization [106]. These data support the role of peripheral CGRP release in SD induced neuronal sensitization.

#### Central inflammatory, electrophysiological, morphological and metabolic changes

##### Cortical inflammation

Although there have been conflicting reports [107], many investigators have observed changes in cortical inflammatory markers after SD in in vivo rodent models [108–110]. Results of PCR and microarray analysis show changes in expression of cytokines, chemokines, and cell adhesion molecules. *Interleukin-1β* (*IL-1β*), *IL-6* and *vascular cell adhesion molecule-1* (*VCAM-1*) are reported to increase at 2 h and 50 h [108], *chemokine (C-C motif) ligand 2* (*CCL2*) and *intercellular cell adhesion molecule-1* (*ICAM-1*) are reported to increase at 3 h [109], and *tumor necrosis factor-α* (*TNF-α*) is reported to increase at 4 h [110] after SD. Recently, we also measured cortical *IL-1β, TNF-α, CCL2, and ICAM-1* after non-invasive optogenetic induced SD (6 SDs over 1 h). With this new method (refer to “*Novel optogenetic methods to trigger SD*” section for details), we observed an acute increase in expression of proinflammatory markers after SD at cortical tissues at least 1 mm away from induction site (unpublished data). Although most of the studies used repeated SD to study the inflammatory consequences, there have been a few studies showing that a single SD evoked by pinprick could elicit the upregulation of proinflammatory markers or the activation of TVS [32, 36]. In fact, by using the noninvasive optogenetic method, we confirmed that a single SD can increase cortical *IL-1β, TNF-α, and CCL2* in the cortex (unpublished data).

##### Electrophysiological changes

In freely moving rats, SD increased corticocortical evoked responses and induced brain derived neurotrophic factor in the ipsilateral cortical hemisphere, consistent with synaptic potentiation in vivo [111]. In vitro studies have shown similar results. In rat amygdala-hippocampal-cortex slices [112] and thalamocortical brain slices [113] SD altered LTP. The perturbated synaptic transmission induced by SD in these circuits may contribute to non-headache symptoms during migraine attacks.

##### Dendritic spine morphology

SD is associated with marked neuronal swelling and beading of dendritic spines, a consequence of profound tissue hypoxia during oxygen supply-demand mismatch [114]. SD-evoked neuronal swelling and dendritic beading are related to chloride cotransporters, which transport water independent of osmotic forces [115], or Panx1 channels [116]. Neuronal endoplasmic reticulum fission has been noted during SD in dendrites and spines, preceded by a dramatic rise in intracellular Ca^2+^ [117]. In contrast to the mechanism underlying dendritic beading, SD-induced endoplasmic reticulum fission depends on NMDA receptor activation and Ca^2+^/calmodulin-dependent protein kinase II. Correlation of endoplasmic reticulum (ER) continuity restoration after fission with recovery of electrocortical activity suggests that ER dynamics may contribute to neuronal activity depression during SD [117].

##### Blood flow, oxygenation and cerebral metabolism

The intense depolarization of SD leads to massive consumption of energy, glucose, and oxygen, leading to intracellular acidification [3, 118–121] and profound hypoxia of the tissue [114, 122, 123]. At the same time, SD evokes multiphasic cerebral blood flow changes and vasomotor responses in the ipsilateral cortex. These phases are not consistently found in all species and could have multiple variations [122–129]. The cerebral blood flow change may include an initial hypoperfusion (5–30% decrease) which coincides with the DC shift and lasts 5–30 s (phase I), a hyperemic phase (30–250% increase) that coincides with repolarization and lasts for a few minutes (phase II), a variable late increase in flow lasting a few minutes (phase III), and a prolonged oligemia (10–40% decrease, phase IV) [122, 127, 129].

In healthy, well-nourished tissue, as is the case in migraine, the intense transmembrane ionic shifts, cell swelling, and metabolic and hemodynamic responses associated with SD do not cause tissue injury, perhaps due to limited duration of hypoxia. However, when SD occurs in metabolically compromised tissue (e.g., ischemia, hypoxia, hypoglycemia), it can lead to irreversible depolarization, injury, and neuronal death [127, 130, 131]. In human neuroimaging studies, migraineurs were found to have a high incidence of white matter hyperintensities or infarct-like lesions, suggesting an increased cerebral vulnerability to ischemia in migraine-susceptible brains [132, 133]. There are multiple possible explanations. Repeated watershed hypoperfusion caused by SD [114] is one of the prevailing theories, although direct evidence from human studies is lacking. Although SD is known to propagate by continuity of gray matter, SD in slice cultures was found to induce significant loss of myelin integrity and myelin basic protein via inflammation and oxidative stress [134]. Microembolism to small arterioles or penetrating arteries might also contribute to SD and the ischemic-like lesions in the white matter [75, 135].

##### Glymphatic flow

The glymphatic system, a glial-dependent perivascular network, is a newly characterized macroscopic extracellular compartment system that clears waste from the brain parenchyma into paravascular spaces, dural lymphatics, and then cervical lymph nodes [136, 137]. The glymphatic system has recently been linked to sleep and traumatic brain injury [138, 139], both of which are associated with migraine risk. An in vivo two-photon microscopy study demonstrated that SD induces a rapid, near-complete closure of the paravascular space around arteries and veins on the pial surface of the cerebral cortex, while impairing interstitial fluid clearance from the parenchyma into the paravascular space [140]. The SD-induced transient impairment of glymphatic flow may impede the clearance of extracellular excitatory neurotransmitters and inflammatory cytokines following SD [36, 38], and thus sustain headache in patients with migraine.

### Behavioral assessments following SD

Behavioral animal models are vital in translational studies of human diseases. While migraine can be defined clinically, preclinical methods used to study mechanisms of migraine model component features thought to be critical for the pathological generation of a migraine attack. However, awake animal models of SD are scarce. In awake and freely moving rats, SD-evoked blood flow changes are consistent with those identified in anesthetized animals [141], suggesting that SD models in awake animals may be useful for modeling migraine aura. Whether awake SD models could recapitulate migraine headache-like behaviors is not yet known. One study showed that KCl injection but not pinprick over the cortex in freely moving rats induced tactile allodynia of the face and hindpaws, and increased Fos expression within the TNC [142]. However, application of KCl onto the dura, without eliciting SD events, could also elicit cutaneous allodynia and increase TNC Fos staining [142]. Therefore, it seems that sustained activation of trigeminal afferents required to establish cutaneous allodynia may be independent of SD. In free-moving rats, induction of a single SD with topical NMDA evoked freezing behavior and wet dog shakes but not ultrasonic vocalization consistent with pain calls (22–27 kHz), suggesting that SD induces anxiety and fear (possibly via amygdala activation) rather than severe pain [61]. Nevertheless, while cutaneous allodynia and ultrasonic vocalization are not completely synonymous with headache; these studies did not refute the proposed link between SD and trigemonovascular activation observed in anesthetized rats [143]. The behavioral responses to “repetitive SDs” evoked by topical KCl have also been evaluated in studies of awake free-moving rats, which demonstrated that SD could propagate into thalamic reticular nucleus and significantly decrease locomotor activity and induce freezing behaviors [144]. It remains uncertain to what extent these behaviors represent pain. However, taken together these neurobiological disturbances are consistent with the migraine condition in humans. While animals cannot be queried about whether or not they have a migraine, these SD associated pathological consequences would suggest that SD is functionally important for the symptomatology of a migraine attack in those that have migraine with aura.

Using the mouse grimace scale [145] it was shown that topical 1 M KCl induced painful craniofacial expression in mice [36]. Although 1 M KCl would readily induce SD in mice, it might also cause significant chemical irritation to the dura and cortex. The newly developed non-invasive optogenetic methods (see above) might circumvent this shortcoming and better address the link between SD and headache. Awake FHM1 R192Q and S218 L mutant mice, exhibit behavioral changes suggestive of spontaneous unilateral head pain, including increased amount of head grooming with unilateral oculotemporal strokes and increased blink rates with one eye closed, induced by novelty and/or restraint stress. In addition to potential signs of head pain, FHM1 mice displayed signs of photophobia [122].

### SD as a model of recurrent episodic or chronic migraine

Migraine is a repetitive neurological attack of disabling headache accompanied by sensory and gastrointestinal disturbances. The classification criteria for migraine takes into account its recurrent nature [146]. Chronic migraine is an implacable incapacitating form of migraine characterized by very frequent attacks. However, the ability to model the recurrent nature of episodic migraine and the very frequent attacks of chronic migraine is a challenge [147]. Despite SD being one of the most widely used models of migraine; using SD to model recurrent episodic or chronic migraine has been hampered by the invasive nature of prior SD models, which often resulted in a barrage of SDs. Injurious methods involving pinprick trauma or direct continuous topical KCl application require placement of a burrhole and likely produce meningeal damage and irritation as part of the surgical preparation. Furthermore, the barrage of SDs occurring at a frequency of 9~12 per hour does not align well with the experience of migraine aura, which would likely be the result of a single SD event. These represent just a few of the challenges involved in using SD to model recurrent or chronic migraine.

There have been only a few preclinical studies on migraine chronification and associated phenotypic behaviors. In one method, the epidural surface or cortex is exposed after scalp reflection and burrhole drilling through the skull. Two methods of chronic daily SD lasting 1–2 weeks were employed. In the first method, a cotton ball soaked in 1 M KCl is placed on the epidural surface for 1 min, followed by a saline wash, to induce a single CSD. In the second method, Tungsten stimulation electrodes are implanted 1-mm below the cortical surface. One second square pulse direct bipolar cathodal stimulation (100–8000 μC) is delivered until a single SD is elicited. Between stimulations, the animals are re-sutured to mark the sites where epidural application of KCl and electrical stimulations were made. An increase in astrocyte staining and decrease in SD susceptibility were observed with these techniques [148]. In a variation of this technique, a 2-mm burrhole was drilled through the skull, taking care to leave the dura intact. A plastic tube (2.5 mm inner diameter) was then affixed to the skull surrounding the burrhole with dental acrylic. The tube was capped to keep the dura moist. Through this tubing, 10–100 mM NMDA or 1–3 M KCl solution (10–20 μl) was allowed to diffuse to the cortical surface below and produce an SD [61]. Using these methods, it is possible to examine the effects of repeated SD on freezing behavior, periorbital mechanical allodynia, and anxiety behaviors [149]. One potential limitation of these techniques is it still involves the potential direct stimulation of the meninges with burrhole drilling and direct application of supraphysiological concentrations of NMDA and KCl directly onto the meningeal surface. Although SD is produced, it is unclear whether the observed changes are due to the SD itself or the disturbance of meningeal nerve terminals.

An optogenetic approach offers the opportunity not only to produce SD noninvasively but also to do so repeatedly [83]. In our laboratory, we have constructed two methods for repeated single event SD induction using optogenetics. In the first approach, a glass coverslip is fixed to the intact skull after a single scalp incision [81]. The durability of the glass coverslip allows daily blue light stimulation (470 nM) for up to 2 weeks. In the second approach, two 10-μL plastic pipette tips cut to a 5-mm length are glued to the intact skull overlying the stimulation site, through which an optical fiber can contact the skull, and the recording site, through which a laser doppler fiber can be placed. SDs are then detected by a characteristic change in the laser doppler flow signal following light stimulation. After SD induction, the fibers are removed, and the animals can be returned to their cages until the next stimulation. This procedure can be done repeatedly in both line 9 and line 18 Thy1-ChR2 YFP transgenic animals. However, we observed an increase in SD threshold with repeated stimulation in this latter method, which may become prohibitive in the line 9 animals as compared to the line 18 animals, as the line 18 animals tend to have lower thresholds (data not published).

These methods can be used to examine changes in pain behavior, anxiety, and cognition as well as changes in light sensitivity and social interactions free of the confounding factors of the invasive induction paradigms previously employed. The use of repeated, non-invasive, optogenetically-induced SD may be able to help answer important questions about the sensory, psychiatric, and cognitive dysfunctions that can accompany chronic migraine. Given the differences in life span of rodents as compared to humans, it is unclear if a direct correlation can be made between the frequency of attacks in humans and those experimentally produced in mice. In that sense, the model is used to examine the nature of change that occurs with repetitive single event less invasive SD but does not (and likely cannot) perfectly recapitulate the human condition of migraine in timing and frequency.

### In-vitro models of SD

In vivo models can be challenging and time consuming due to microsurgical preparation and maintenance of stable systemic physiological conditions under anesthesia. Nevertheless, they are essential for preclinical therapeutic testing. In vitro models in brain slices or chicken retina are also critical in SD research [150–152]. The key advantage of the brain slice over a whole animal preparation is that parameters such as temperature, oxygenation, pH, ionic, and pharmacological environment can be precisely controlled. Cellular resolution imaging and high-quality electrophysiological recordings can be better performed in a slice than in vivo. Slice preparations also allow access to brain regions that are difficult to access in in vivo studies, especially in human brains. In vitro SD studies in chicken retina, which has characteristics similar to brain slices, also allowed systemic evaluation for SD pharmacology [151]. However, these in vitro models are not networked nervous system. To understand the complex brain circuitry involved in migraines, the information obtained from in vitro models is limited.

## Conclusion

SD is a validated experimental model of migraine aura. Studies on the physiological, biochemical, and behavioral consequences of SD have aided in understanding the complex pathobiology of migraine and could present viable targets for novel migraine therapeutics. With the refinement of models and advancement of techniques, such as miniaturized wireless implants, remote telemetry, and noninvasive optical imaging, more SD studies in awake animals (even in gyrencephalic brains) can be expected. In combination with genetic editing tools, optogenetics, chemogenetics, tissue clearing, and other tools for functional circuit mapping, next-generation SD models will be instrumental to solving the many remaining questions in migraine research.

## Abbreviations

BBB: Blood-brain barrier
FHM: Familial hemiplegic migraine
SD: Spreading depression
TNC: Trigeminal nucleus caudalis
TVS: Trigeminovascular system
